# AKP-11 - A Novel S1P1 Agonist with Favorable Safety Profile Attenuates Experimental Autoimmune Encephalomyelitis in Rat Model of Multiple Sclerosis

**DOI:** 10.1371/journal.pone.0141781

**Published:** 2015-10-29

**Authors:** Devadoss J. Samuvel, Nishant Saxena, Jasdeep S. Dhindsa, Avtar K. Singh, Gurmit S. Gill, Damian W. Grobelny, Inderjit Singh

**Affiliations:** 1 Charles P. Darby Children’s Research Institute, Department of Pediatrics, Medical University of South Carolina, Charleston, South Carolina, United States of America; 2 Department of Pathology and Laboratory Medicine, Medical University of South Carolina, Charleston, South Carolina, United States of America; 3 Akaal Pharma Pty Ltd., 310E Thomas Cherry Building, Bundoora, Australia; Hanyang University, REPUBLIC OF KOREA

## Abstract

Sphingosine-1-phosphate receptor 1 (S1P1) mediated regulation of lymphocyte egress from lymphoid organs is recognized as the mechanism of FTY720 (Fingolimod, Gilenya) efficacy in relapsing-remitting forms of multiple sclerosis (RRMS). In this study we describe a novel S1P1 agonist AKP-11, next generation of S1P1 agonist, with immunomodulatory activities in cell culture model and for therapeutic efficacy against an animal model of MS, i.e. experimental autoimmune encephalomyelitis (EAE) but without the adverse effects observed with FTY720. Like FTY720, AKP-11 bound to S1P1 is internalized and activates intracellular AKT and ERKs cellular signaling pathways. In contrast to FTY720, AKP-11 mediated S1P1 downregulation is independent of sphingosine kinase activity indicating it to be a direct agonist of S1P1. The S1P1 loss and inhibition of lymphocyte egress by FTY720 leads to lymphopenia. In comparison with FTY720, oral administration of AKP-11 caused milder and reversible lymphopenia while providing a similar degree of therapeutic efficacy in the EAE animal model. Consistent with the observed reversible lymphopenia with AKP-11, the S1P1 recycled back to cell membrane in AKP-11 treated cells following its withdrawal, but not with withdrawal of FTY720. Accordingly, a smaller degree of ubiquitination and proteolysis of S1P1 was observed in AKP-11 treated cells as compared to FTY720. Consistent with previous observations, FTY720 treatment is associated with adverse effects of bradycardia and lung vascular leaks in rodents, whereas AKP-11 treatment had undetectable effects on bradycardia and reduced lung vascular leaks as compared to FTY720. Taken together, the data documents that AKP-11 treatment cause milder and reversible lymphopenia with milder adverse effects while maintaining therapeutic efficacy similar to that observed with FTY720, thus indicating therapeutic potential of AKP-11 for treatment of MS and related autoimmune disorders.

## Introduction

Multiple Sclerosis (MS), is a chronic inflammatory autoimmune disorder of the central nervous system that leads to chronic demyelination with axonal damage and neuronal loss [1]. MS is a major cause of disability among young adults with a worldwide incidence estimated to be 2.5 million [2]. It is initiated with central nervous system (CNS) infiltration of autoreactive T cells and macrophages with expression of their pro-inflammatory effector functions (TNFα, IL-1β, IFN-γ and IL-17), leading to demyelination, degeneration and lesion formation. The etiology and pathology of this disease are still unclear. In laboratory animals, MS is modeled as an experimental autoimmune encephalomyelitis (EAE), either by subcutaneous immunization with various myelin proteins (MBP; myelin basic protein, PLP; proteolipid protein or MOG; myelin oligodendrocyte protein), or by the adoptive transfer of encephalitogenic T cells [3–5]. Cytokine mediated immune response is one of the important factors in MS pathology [6,7]. Current FDA approved immunomodulatory drugs provide limited efficacy as the CNS disease progression continues. This underscores the need for greater understanding of the pathobiology of the MS disease process, and also the identification of drugs that target the clinical disease process including immune responses as well as the manifest CNS disease. Available therapies include interferon β, glatiramer acetate, natalizumab and mitoxantrone, which are reported to be disease modifying drugs for relapsing-remitting MS (RRMS) progression. Glucocorticoids, namely 6α-methylprednisolone (6-MP) or prednisone [8], are acute stress response hormones known to act on many biological systems that reduce MS symptomology [9]. These drugs are injected and act on the immune system with relatively little effect on the CNS [10–13].

Fingolimod (FTY720, Gilenya), a derivative of the fungal metabolite myriocin, is a structural analogue of endogenous lysolipid sphingosine and is the first oral immunomodulatory drug that received FDA approval for the treatment of RRMS [14–16]. It is a prodrug that acts by modulating sphingosine 1 phosphate receptor 1(S1P1) signaling following the conversion of its phosphorylated form [17]. In vivo, FTY720 is converted to phosphorylated derivative FTY720-P by sphingosine kinase-2 [17,18]. FTY720P having structural resemblance to sphingosine-1- phosphate (S1P), interacts with the G protein coupled receptor of S1P (S1P1, 3, 4 and 5), inducing downstream cellular responses in various cell systems [14,17,19]. The S1P1-5 are expressed by multiple cell types and are predominantly expressed on immune cells and CNS and fingolimod has high affinity for S1P1 [20]. The efficacy of fingolimod against MS is mediated by its functional antagonism of S1P1 which reduces the egress of autoreactive lymphocyte from lymphoid organs [21,22]. The role of S1P1 signaling in lymphocyte egression from secondary lymphoid organs is demonstrated by S1P1 deletion in hematopoietic cells and S1P1 agonists [19,22,23]. FTY720P causes long-lasting internalization and degradation and thus loss of S1P1 receptor, thereby blocking S1P signaling mediated lymphocyte egress [24]. The reduced egress of reactive lymphocytes leads to reduced infiltration of autoreactive lymphocytes into CNS leading to suppression of the immune response [14] in MS patients. Therefore, the efficacy of FTY720 is attributed to the induction of peripheral lymphopenia [25–27]. However, expression of S1P1 in multiple cell types for respective functions [28] and very slow reconstitution of circulating lymphocyte count following discontinuation of FTY720, results in associated adverse effects of bradycardia [29] and lung vascular dysfunction [30,31]. This points to concern that sustained lymphopenia in management of patients with long term use of FTY720 [32]. These studies suggest that drugs targeting S1P1 mechanisms without the adverse effects will be highly desirable.

In this study, we investigated the efficacy of a novel S1P1 agonist, using in vitro cell culture and animal model of EAE. In a direct comparison, oral administration of AKP-11 and FTY720 to EAE were equally effective in attenuation of clinical disease. The downregulation of S1P1 receptor by AKP-11 is independent of the sphingosine kinase mechanism. Although treatment with both FTY720 and AKP-11 decreased the peripheral lymphocytes, the reduction was much greater in FTY720 treated animals than those treated with AKP-11. Furthermore, AKP-11 treatment caused milder and reversible lymphopenia as compared to FTY720. The reconstitution of circulating lymphocytes was much quicker at 48hrs in AKP-11 treated animals than FTY720 treated animals at 48hrs following cessation of the drug treatments. Accordingly, AKP-11 treatment compared to FTY720 had much milder effects of bradycardia and pulmonary vascular dysfunction. Collectively, these data provide evidence that AKP-11 has potent immune modulatory activity for treatment of EAE/MS, but with relatively low adverse effects suggesting AKP-11 as a potential therapeutic drug for MS patients.

## Materials and Methods

### Ethics Statement

Adult female Lewis rats weighing 200-230g were purchased from Charles River laboratory (Wilmington, MA) and housed in the animal care facility at the Medical University of South Carolina (MUSC) throughout the experiment and provided with food and water *ad lib*. All animal experiments were conducted in accordance with accepted standards of humane care, as outlined in the ethical guidelines and approved by MUSC's Animal Ethics Committee. When rats were paralyzed in EAE induction procedure, they were provided with hydrogel (Clear H2O) and/or moistened food in the cage and rats were monitored daily by both researchers and veterinarians. At the time of termination of experiments, rats were sacrificed under deep anesthesia with ketamine and xylazine. None of the rats reached moribundity during the studies.

### EAE induction

EAE was induced as described previously [33]. Briefly, rats were anesthetized with ketamine and xylazine and were immunized in the hind of foot pad with 25 μg guinea pig myelin basic protein (MBP) (Sigma, St Louis, MO) emulsified (1:1) in 100 μL complete Freund’s adjuvant on day 0 and day 7 in incomplete Freund’s adjuvant. Additionally, 200 ng of Pertussis toxin (Sigma, St Louis, MO) was given on day 0 and day 1 by i.p. injection. EAE clinical symptom was monitored daily and was graded according to the following common scale 0–5: 0, no clinical signs; limp tail or waddling gait with tail tonicity, 1; waddling gait with limp tail (ataxia), 2; ataxia with partial limb paralysis, 2.5; full paralysis of one limb, 3; full paralysis of one limb with partial paralysis of second limb, 3.5; full paralysis of two limbs, 4; moribund stage, 4.5; and death, 5 [34,35]. After the onset of the disease, when the animals reached clinical score at 2, they were given orally with AKP-11 or FTY720.

AKP-11 was synthesized and supplied by Akaal Pharma LLP, Norbury, London (PCT application W0-2010-043000A1). FTY720 was obtained from Cayman Chemical, Ann Arbor, Michigan, USA. Molecular weight of AKP-11 and FTY720 are 443.5 and 343.9 respectively.

### Cell Culture and S1P1 transfection

CHO cells were obtained from ATCC (American Type Culture Collection, Manassas, VA) and cultured in Dulbecco's modified Eagle's medium (high glucose) supplemented with 10% FBS and antibiotics (Invitrogen). For stable transfection, 35-mm dishes of CHO cells at the density of 1.5 X 10^5^ cells were transfected with 2μg of human S1P1 (Missouri S&T cDNA Resource Centre) by using Lipofectamine2000 according to manufacturer’s instructions (Invitrogen). Stable cells were selected after thirty six hours post-transfection by treatment with 800 μg/ml geneticin in the medium for 2 weeks. After selection, they were maintained in the DMEM medium containing 10% charcoal FBS and 400μg/ml geneticin (Invitrogen). Before treatment with AKP-11 or FTY720 or FTY720P (Echelon Biosciences Inc. Salt Lake City, UT), the cells were serum starved in six well plates for 4hrs at 37°C and then they were incubated with the above compounds at 37°C.

### Cell Surface Biotinylation

After drug treatment the cells were washed with PBS and kept on ice. For membrane protein detection, the cells were labeled with Sulfo-NHS-SS-biotin following manufacturer’s instructions (Pierce, Rockford, IL). Briefly, cells were incubated with membrane-impermeable sulfo-NHS-SS-biotin (1mg/ml) at 4°C for 45min. After the incubation, the cells were washed with PBS and the unbound biotin was quenched with 100mM glycine in PBS at 4°C for 10 min. Cells were lysed with RIPA buffer (Pierce, Rockford, IL) and incubated with NeutraAvidin beads (Pierce, Rockford, IL) overnight at 4°C. Then next day they were centrifuged, washed three times with RIPA buffer and the bound proteins were eluted from the NeutraAvidin beads by boiling with 2X sample buffer.

### Real time PCR Analysis

Total RNA was isolated from spinal cord tissues using TRIzol reagent. The first-strand cDNA was synthesized using the iScript^TM^ cDNA synthesis kit (Bio-Rad, Hercules, CA, USA) according to the manufacturer instructions. Quantitative PCR for various genes were determined using a SYBR green Super mix (Bio-Rad). Rat specific primer for CD4 was purchased from Qiagen (Gaithersburg, MD). Real time PCR was run in the iCycler™ real time detection system (Bio-Rad) and thermal cycling parameters were as follows: activation of iTaq DNA polymerase at 95°C for 10 min, followed by 40 cycles of amplification at 95°C for 30 sec and 60°C for 1 min. A melt curve analysis was performed for specificity of real-time PCR. Quantification of mRNA was calculated using the starting quantity of the cDNA of interest relative to that of GAPDH cDNA in the same sample.

### Lymphocytes counts in blood

Before cardiac blood collection in each experiment group, rats were anesthetized with ketamine and xylazine and the whole-blood samples (200 μL) were collected into EDTA blood collection tubes (BD Biosciences) and all blood cell counts were measured by an automated hematology analyzer. For lymphocyte subsets, 50 μL of whole blood was mixed with staining buffer (20 μL) containing fluorescently labelled antibodies (15 min, RT) and RBC were lysed with FACS lysing solution (BD Biosciences) prior to FACS analysis. Antibodies used for staining were from BD Pharmingen and included APC-labelled anti-rat CD3 (clone 1F4), FITC-labelled anti-rat CD4 (clone OX-38) and PE-labelled anti-rat CD8 (clone OX-8) and PE-labelled anti-rat CD62L (clone HRL1) and appropriate isotype-matched controls.

### Histological Analysis

EAE animals and control animals were anesthetized and they were perfused first with saline and then 4% paraformaldehyde and spinal cords were fixed in 10% formalin. To assess infiltration of T cells, H&E stain was performed in paraffin-embedded, 4-μm-thick transverse sections of spinal cord. For assessing demyelination, Luxol Fast Blue was done. Pictures were taken using an Olympus Compound Microscope (Olympus BX-60) with digital camera attached.

### ELISA and Western blot analysis

For quantification of cytokines, ELISA was carried out from the spinal tissue samples. They were homogenized with PBS and they were normalized with protein. Protein was estimated by DC method (Bio-rad). IFN-γ and IL-10 ELISA kit were purchased from R&D systems (Minneapolis, MN) and IL-17A from Biolegend (San Diego, CA).

For western blot, tissues were lysed in RIPA buffer containing protease and phosphatase inhibitors and the lysates were spun at 15,000g for 30 min at 4°C. The resulting supernatant protein was estimated by DC method and used for western blot. Proteins were separated by 4–20% SDS gels and transferred to a nitrocellulose membrane (GE Healthcare Life Sciences, Arlington Heights, IL). The membrane was blocked with 5% nonfat milk in Tris-buffered saline containing 0.05% Tween for 1 hr. at room temperature. For phospho-protein, instead of non-fat milk 5% BSA was used. The membranes were incubated overnight at 4°C with primary antibodies at 1:1000 dilutions in blocking buffer (TTBS with 2% nonfat milk). The following primary antibodies were used in the present study; Ubiquitin, MBP (Santa Cruz, Dallas, TX), Phospho AKT and ERK, Total AKT and ERK, β-actin (Cell Signaling, Danvers, MA) and NF-200, HA (Covance, MA). After washing, the membranes were incubated with 1:10,000 diluted horseradish peroxidase conjugated secondary antibody (Jackson Immunoresearch Lab, West Grove, PA) for 1hr at room temperature, again washed, reacted with ECL reagent (GE Healthcare Life Science, Pittsburgh, PA), and were exposed to ECL film. Autoradiographs were scanned and the band intensity was quantified by NIH J image.

### Isolation and flow cytometric analysis of mononuclear cells from spinal cord

Mononuclear cells were isolated from the spinal cords of immunized rats on day 26 following immunization. After perfusion with sterile PBS, the spinal cord was dissociated by passing through a 70μm cell strainer and centrifuged at 500g for 5min. The pellet was re-suspended on 30% precool (Sigma Aldrich), and subjected to discontinuous 30%/70% percoll gradient at 800g for 20 min. Mononuclear cells were removed from the interphase, washed and suspended in RPMI culture medium. The cells were stimulated with cell stimulator plus protein transporter inhibitor (eBioscience, San Diego, CA) for 4hrs and were surface stained with CD4 antibody. The cells were then fixed and permeabilized (permeabilization buffer, BD biosciences), followed by staining with IL-17A (ebiosciences).

### *In vitro* Th17 generation

CD4^+^T cells isolated from the spleen using MagCellet^TM^ Rat CD4^+^ T cell isolation kit (R&D Systems) were stimulated with anti-CD3 and CD28 antibodies (Biolegend) under Th17 differentiation conditions (recombinant IL-6, 20 ng/mL; recombinant TGF-β, 3 ng/mL; anti-IL-4, 10 ng/mL; and anti–IFN-γ, 10 ng/mL; Biolegend) in the absence or presence of AKP-11 or FTY720 for 3 days. The culture supernatants were collected and measured for Th17 (IL-17) cytokine production by ELISA.

### Immunofluorescence

Stable cells expressing S1P1 CHO were seeded on four well chamber slides (Fisher Scientific, Pittsburgh, PA) and after 24 hours they were starved in serum free conditions. They were then incubated with AKP-11 or FTY720 or FTY720P for 1hr and after incubation washed with PBS and fixed with 4% paraformaldehyde. The cells were permeablised with 0.2% Triton for 5 min and pre-blocked with 5% BSA for 1hr at room temperature. They were then incubated with HA antibody for overnight at 4°C and next day washed with PBS and incubated with Alexa Fluor 488 fluorophore conjugated secondary antibody (Molecular Probes, Invitrogen, CA) for 1hr. Afterwards they were washed with PBS and nuclei were stained with Hoechst (Fisher Scientific, Pittsburgh, PA). Finally they were mounted with mounting medium (Vector Lab, Burlingame, CA) and visualized under Olympus FLUOVIEW FV1000 confocal microscope.

### Lung permeability assay

Lung permeability changes were measured by Evans blue dye (EBD, Sigma Aldrich, and St. Louis, MO) leakage after administration of AKP-11 and FTY720. AKP-11 1.3mg/kg and FTY720 1mg/kg were administered orally and after 24hrs, EBD (0.5% in saline) was administered by tail vein injection. Two hours later, the animals were bled by cardiac puncture and pulmonary vasculature was perfused with saline to remove EBD from the vascular spaces. Lungs were removed, photographed and dried at 60°C for 24hrs. They were then weighed and EBD was extracted in dimethylformamide (Sigma Aldrich, St. Louis, MO) at 37°C for 24hrs and quantitated spectrophotometrically at 620 and 740 nm.

### Heart rate and blood pressure measurement

Heart rate and blood pressure were measured non-invasively in rats by Coda Tail-Cuff Blood Pressure system (Kent Scientific, USA). Rats were administrated orally with 1.3 mg/kg AKP-11, 1 mg/kg FTY720 and vehicle. After post administration, heart rates were measured at 1, 2, 4, 6, 12, and 24 hr. The mean blood pressure was calculated as follows: Mean blood pressure = (systolic blood pressure—diastolic blood pressure)/3 + diastolic blood pressure.

### Statistical analysis

Statistical analysis was performed using Graph pad Prism 5.0 software. Significance between the groups were determined by one-way analysis of variance (ANOVA) followed by Tukey’s post-hoc test. The *p-*value less than 0.05 were considered statistically significant.

## Results

### AKP-11 attenuates EAE disease

**Fig 1:**
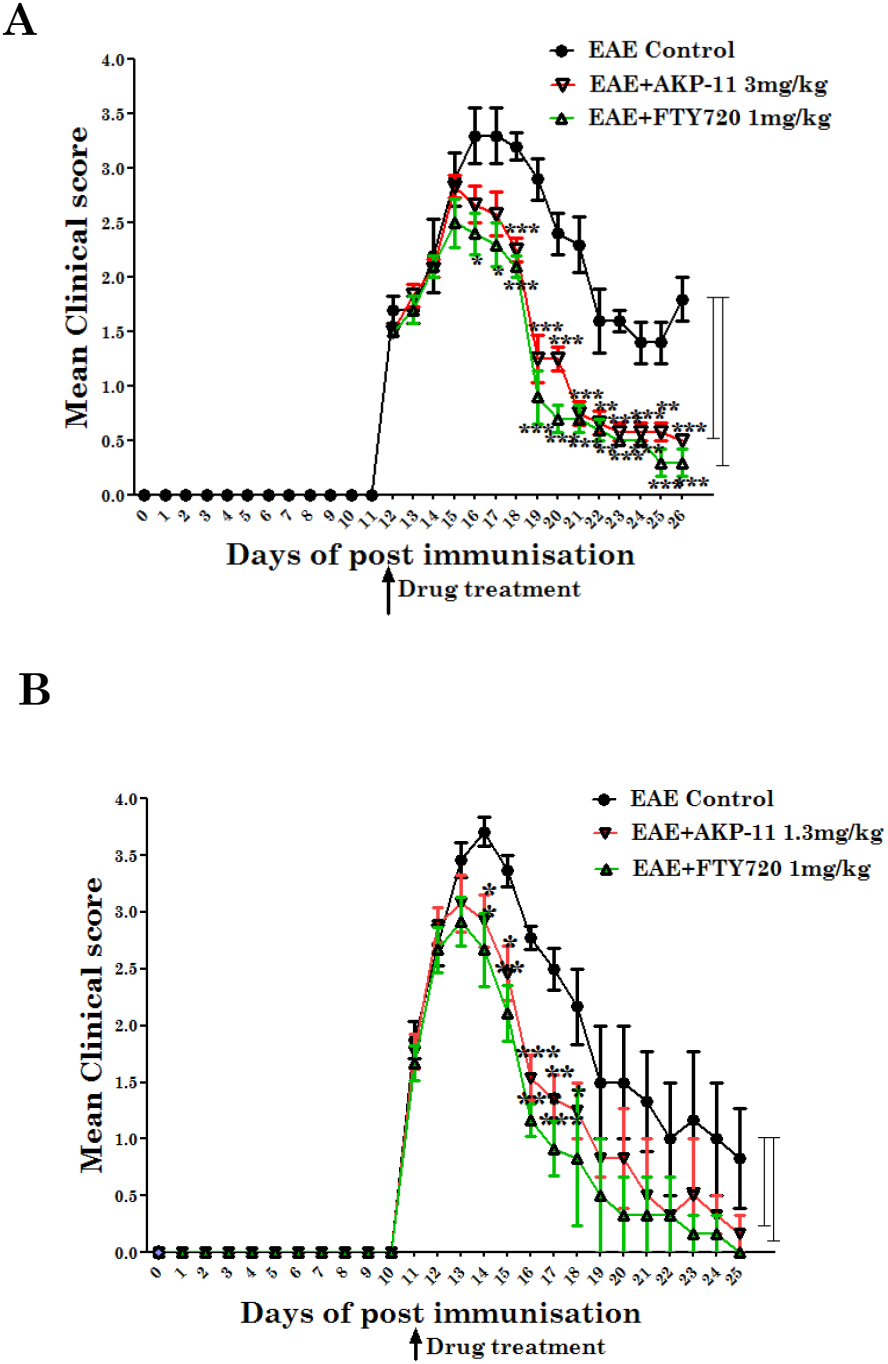
AKP-11 and FTY720 treatments attenuate EAE disease in the Lewis rat. (A-B) EAE was induced in female Lewis rats with guinea pig MBP (25μg/rat). EAE developed rats were divided into 3 groups on day 11 or 12 after immunization and administered vehicle or AKP-11 (3 or 1.3 mg/kg) or FTY720 (1 mg/kg) orally every-day until day 26. Clinical scores: 0, no clinical signs; limp tail or waddling gait with tail tonicity, 1; waddling gait with limp tail (ataxia), 2; ataxia with partial limb paralysis, 2.5; full paralysis of one limb, 3; full paralysis of one limb with partial paralysis of second limb, 3.5; full paralysis of two limbs, 4; moribund stage, 4.5; and death, 5. Data represents mean ± SEM of three independent experiments (6 animals per group). Statistical significance is indicated as *p<0.05 **p<0.01 and ***p<0.001.

EAE is the animal model of MS which is extensively used for investigating clinical studies for MS drugs [36]. Attenuation of EAE disease by an oral drug FTY720 as a S1P1 agonist was also studied in the EAE animal models [37–39]. Here, we investigated the relative efficacy of two S1P1 agonists AKP-11 and FTY720 in rodent model of EAE disease. Both FTY720 and AKP-11 were orally administered starting at the onset of clinical disease (score of EAE at 2.0) as described under methods and material. Both FTY720 and AKP-11 treatments attenuated the EAE disease (p<0.001) thus documenting therapeutic efficacy against EAE disease (Fig 1A and 1B). The observed relative similar efficacy of AKP-11 (1.3mg/kg) and FTY720 (1mg/kg) indicates that both compounds are practically equally effective against the EAE disease.

### FTY720 and AKP-11 treatments decreased peripheral lymphocyte count in both control and EAE animals

**Fig 2:**
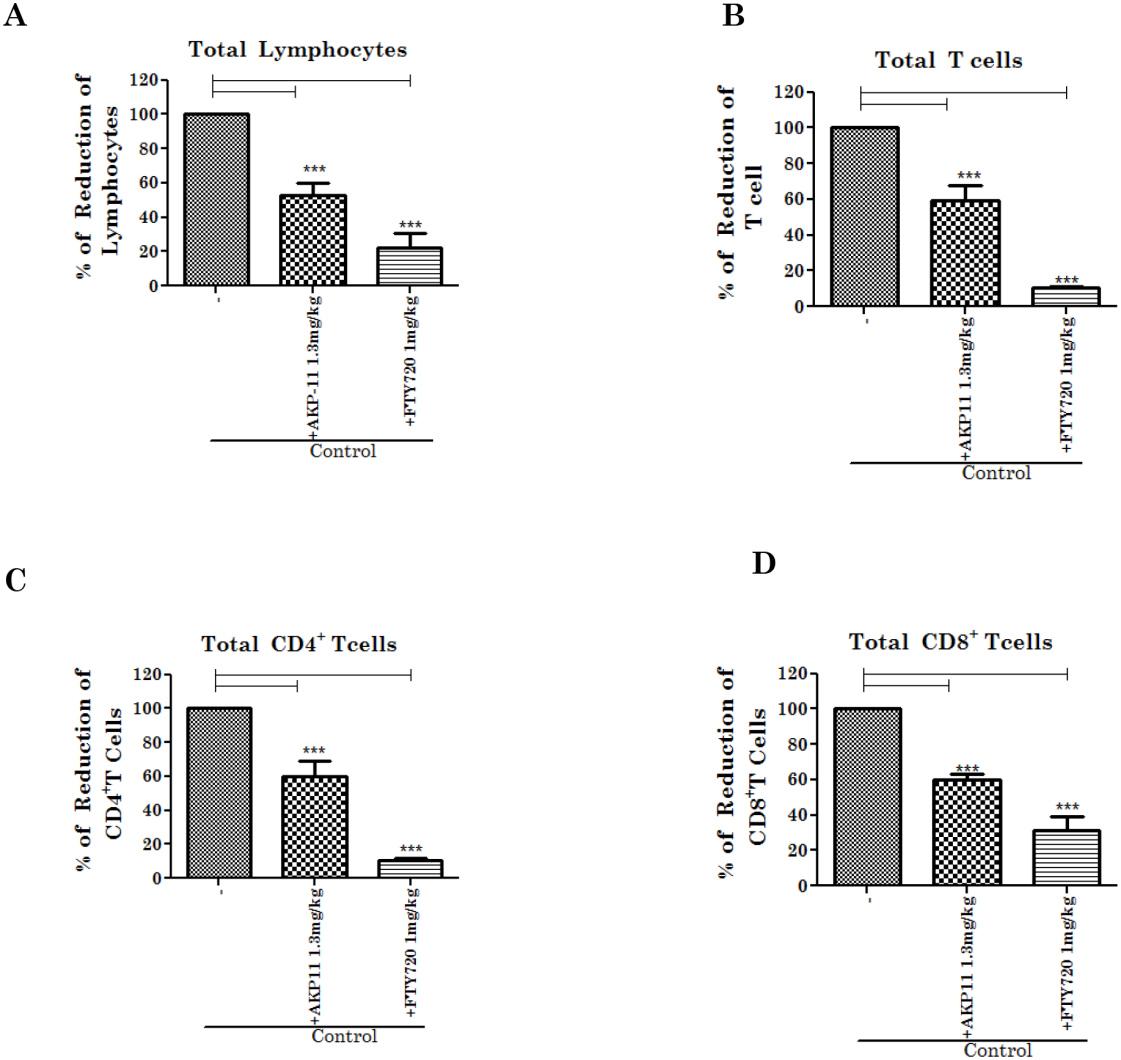
AKP-11 and FTY720 treatments reduce PBL count. Control animals were treated with a single dose of AKP-11 (1.3mg/kg) or FTY720 (1mg/kg) and blood lymphocyte counts were analyzed by flow cytometry after 24 hrs. (A) Total lymphocytes, (B) Total T lymphocytes, (C) Total CD4^+^T lymphocytes, (D) Total CD8^+^T lymphocytes populations were reduced in AKP-11 and FTY720 treated animals compared to vehicle control animals. (Total lymphocytes 100% of control 2800 cells/μL, T cells 100% of control 2000 cells/ ul, CD4^+^T cells 100% of control 1400 cells/ μL, CD8^+^T cells 100% of control 600 cells/μL). Data represents mean ± SEM of three independent experiments (6 animals per group). Statistical significance is indicated as *p<0.05 **p<0.01 and ***p<0.001.

**Fig 3:**
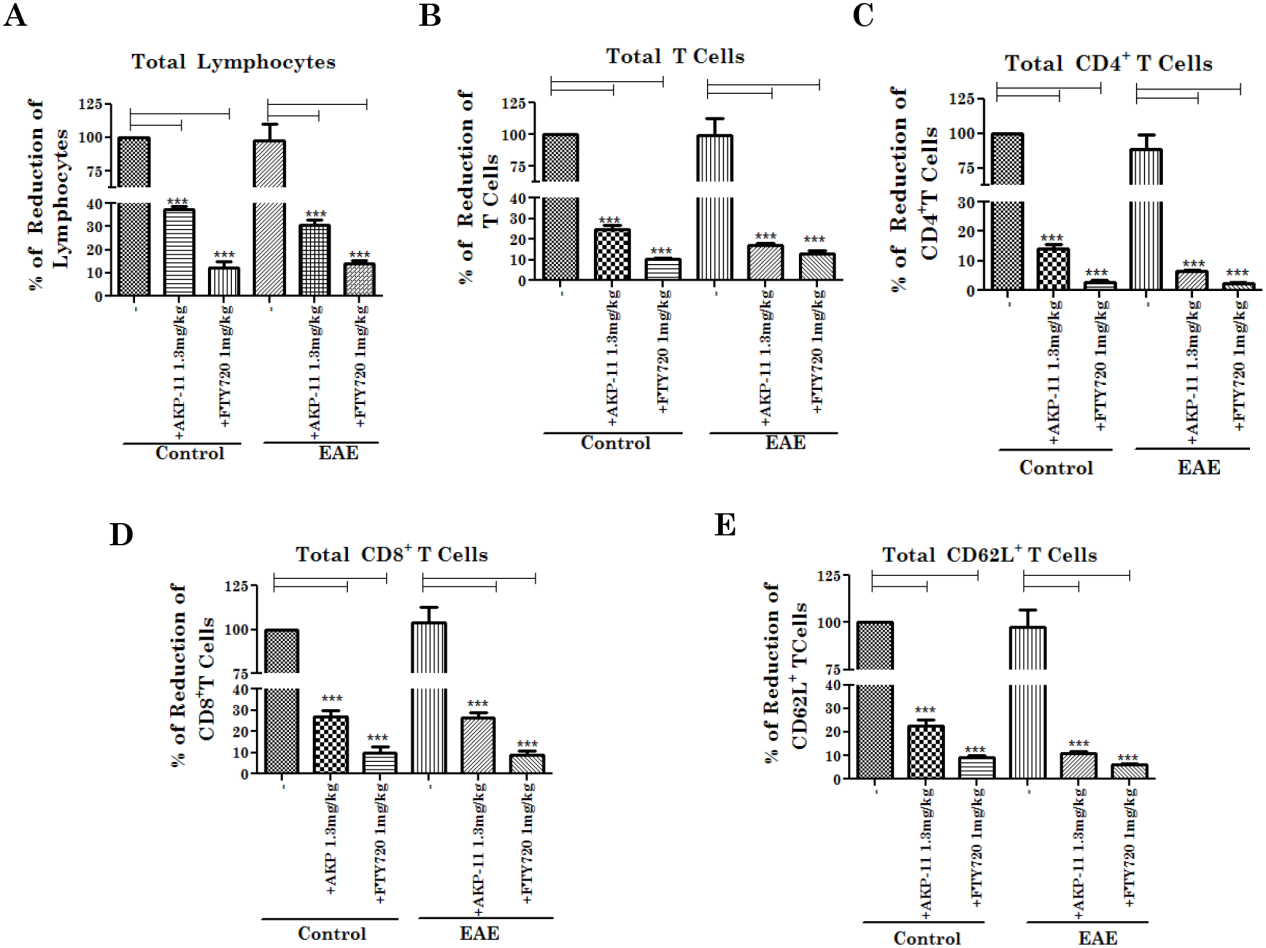
AKP-11 and FTY720 reduce PBL count in rats during EAE. Control and EAE animals were treated with AKP-11 (1.3mg/kg) or FTY720 (1mg/kg) for 7 days and blood lymphocyte subsets were analyzed by flow cytometry after 24 hrs. of last drug treatment. (A) Total lymphocytes, (B) Total T lymphocytes, (C) Total CD4^+^T lymphocytes, (D) Total CD8^+^T lymphocytes (E) Total CD62L^+^T lymphocytes populations were reduced in AKP-11 and FTY720 treated animals compared to vehicle control animals. (Total lymphocytes 100% of control 2800 cells/μL, T cells 100% of control 2000 cells/μL, CD4^+^T cells 100% of control 1400 cells/μL, CD8^+^T cells 100% of control 600 cells/μL and homing receptor CD62L^+^T cells 100% of control 1800 cells/μL). Data represents mean ± SEM of three independent experiments (6 animals per group). Statistical significance is indicated as *p<0.05 **p<0.01 and ***p<0.001.

**Fig 4:**
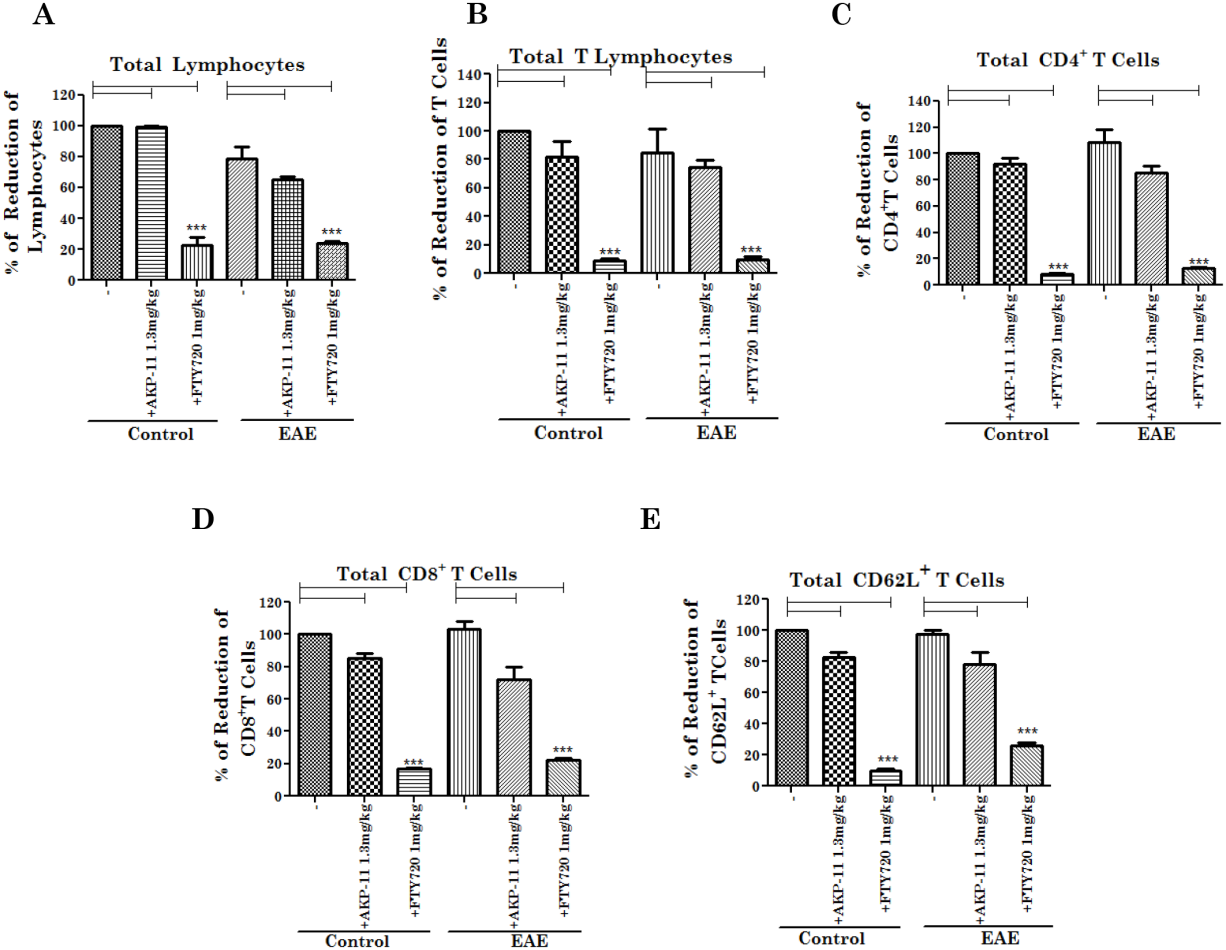
Comparison of AKP-11 and FTY720 on reversal of lymphopenia in control and EAE animals with different drug treatments followed by withdrawal for 48hrs. Control and EAE animals were treated with AKP-11 or FTY720 for 14 days and blood lymphocyte subsets were analyzed after 48hrs of discontinue of drug treatment. (A) Total lymphocytes, (B) Total T lymphocytes, (C) Total CD4^+^T lymphocytes, (D) Total CD8^+^T lymphocytes, (E) Total CD62L^+^T lymphocytes populations were reversed to normal levels only in AKP-11 not in FTY720 treated animals. (Total lymphocytes 100% of control 2800 cells/μL, T cells 100% of control 2000 cells/μL, CD4^+^T cells 100% of control 1400 cells/μL and CD8^+^T cells 100% of control 600 cells/μL and homing receptor CD62L^+^T cells 100% of control 1800 cells/μL). Data represents mean ± SEM of three independent experiments (6 animals per group). Statistical significance is indicated as *p<0.05 **p<0.01 and ***p<0.001.

FTY720 [26,37] and other S1P1 receptor agonists [39–41] are known to cause lymphocyte sequestration in lymph nodes resulting in reduced peripheral blood count of lymphocytes. It is the mechanism of observed efficacy [14] as well as one of the mechanisms of observed adverse effects of FTY720 medication for MS [42]. Therefore, we investigated the peripheral T lymphocyte count following FTY720 or AKP-11 treatments in EAE and control animals. Fig 2 shows the effects on peripheral lymphocyte count 24hrs following one time treatment of AKP-11 or FTY720. As expected, FTY720 and AKP-11 treatments, as agonists of S1P1, reduced peripheral total blood lymphocyte and T lymphocyte counts significantly (p<0.001) when compared to untreated controls, however, lymphopenia was much severe in animals treated with FTY720 than with AKP-11 (Fig 2). The reduction in total lymphocytes and T cells was 78% and 90% in FTY720 and 48% and 41% in AKP-11 treated animals respectively as compared to untreated controls (Fig 2A and 2B). The reduction in the CD4^+^ and CD8^+^ T cell populations was 90% and 69% in FTY720 and 41% and 40% in AKP-11 treated animals respectively, as compared to untreated controls (Fig 2C and 2D). Accordingly, the reductions were greater in animals treated in FTY720. Next, we investigated the peripheral lymphocyte count following treatment of control and EAE animals with AKP-11 and FTY720 following onset of clinical disease for seven days (Fig 3). FTY720 and AKP-11 treatments of control and EAE animals decreased total lymphocyte, T cells and CD4^+^ T cells and CD8^+^T cells but a greater decrease was observed in animals treated with FTY720 than those treated with AKP-11 (Fig 3). In agreement with the previous reported reduction of CD62L^+^ T cells [43], FTY720 and AKP-11 treatments reduced the levels of CD62L^+^ T cells but with a greater decrease with FTY720 as compared to AKP-11 in both EAE and control animals (Fig 3). Since efficacy of FTY720 is attributed by the induction of lymphopenia, long term use of this drug is a concern in MS patient care because reconstitution of circulating lymphocyte count is reported to lag from weeks to months following drug trial [32]. Therefore, we also evaluated circulating lymphocyte counts following withdrawal in control and EAE animals treated for 14 days with AKP-11 or FTY720 starting at onset of clinical disease. The T cell populations were analyzed following withdrawal of drugs for 48hrs in both EAE and control groups. Interestingly, reconstitution of circulating lymphocytes was much more efficient in AKP-11 treated animals than FTY720 treated animals (Fig 4). Total lymphocyte, CD4^+^, CD8^+^ and CD62L^+^ T cell populations following 48hrs withdrawal of AKP-11 treatment in control and EAE animals were restored to the levels similar to untreated control and EAE animals. On the other hand, withdrawal of FTY720 treatment for 48hrs had relatively little effect on reversal of lymphopenia (Fig 4). The comparison of lymphocyte counts at 24hrs (Fig 3) versus 48hrs (Fig 4) following last drug treatment documents milder and faster reversible (short lasting) lymphopenia with AKP-11 as compared to FTY720 suggesting that milder and short term lymphopenia with AKP-11 treatment may cause milder adverse effects as compared to FTY720.

### AKP-11 treatment reduces CNS infiltration of T cells and Cytokines and enhances neuroprotection

**Fig 5:**
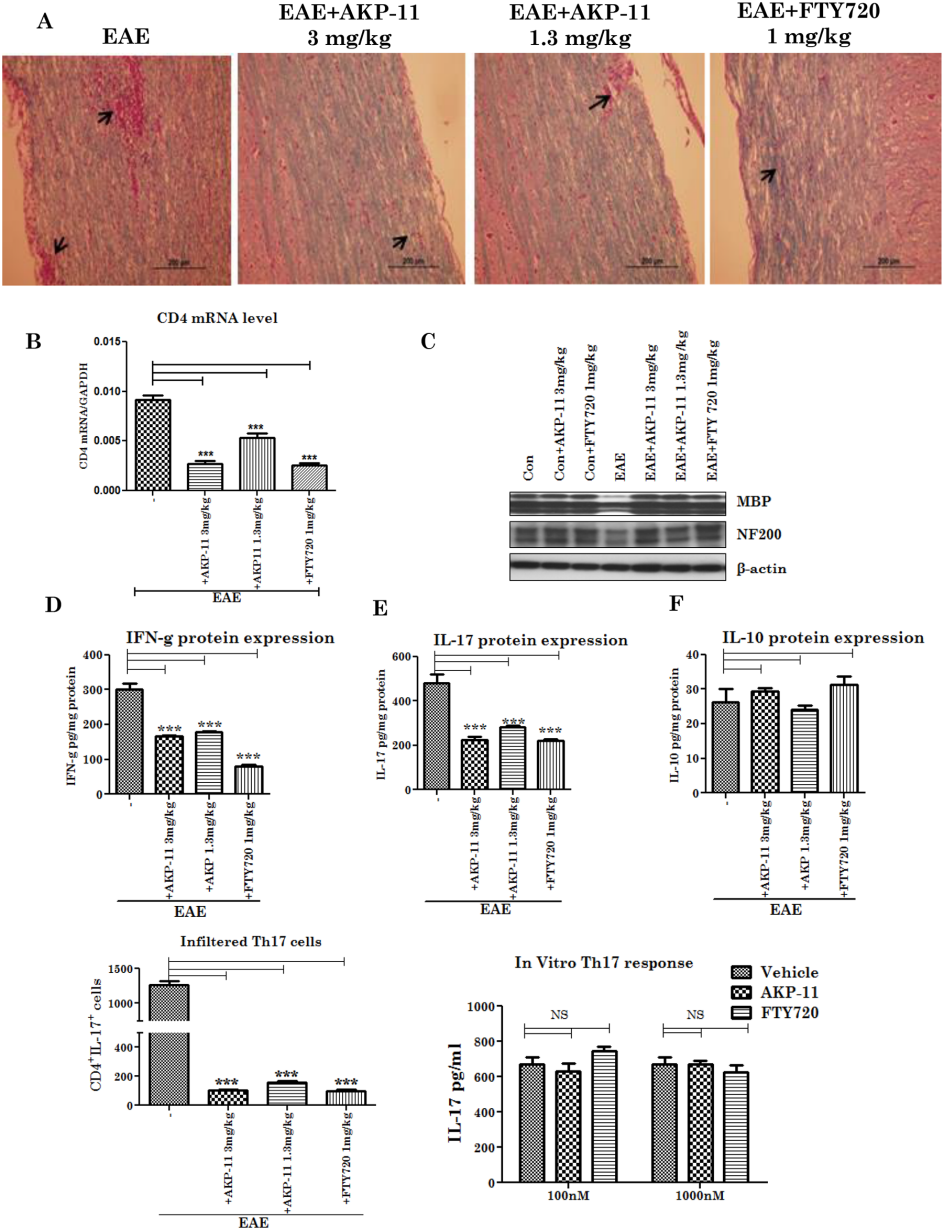
AKP-11 and FTY720 prevent the infiltration of mononuclear cells into the CNS of EAE animals and decrease the inflammatory cytokines and protect the MBP and NF-200. (A) Control and EAE animals were treated with AKP-11 (3 or 1.3mg/kg) or FTY720 (1mg/kg) for 14 days starting onset of clinical disease (remission). Spinal cord tissue was fixed and infiltration of mononuclear cells was examined by H&E staining and for demyelination, Luxol Blue Fast staining was performed. (B) Infiltrated CD4 cells in the spinal cord were analyzed by RT-PCR. (C) Western blotting for MBP and NF-200 in the above tissue samples. (D-F) IFN-γ, IL-17, IL-10 were analyzed with ELISA from spinal cord tissue respectively.(G) Th17 cell population in spinal cord mononuclear cell infiltrates was quantified by flow cytometry. (H) Spleen CD4^+^ T cells were stimulated with CD3 and CD28 antibodies under Th17 conditions in the presence or absence of AKP-11 or FTY720 (100, 1000nM) for 72hrs and IL-17 was measured by ELISA. Data represents mean ± SEM of three independent experiments (6 animals per group). Statistical significance is indicated as *p<0.05 **p<0.01 and ***p<0.001, NS- not significant.

Increased infiltration of T cells, inflammatory cytokines, and demyelination are characteristic features of EAE pathology. FTY720 is reported to reduce the T cell infiltration and inflammatory cytokines in the CNS [27,39]. Next, we investigated the effects of AKP-11 and FTY720 treatments on CNS infiltration of vascular immune cells, expression of pro-inflammatory cytokines and myelin basic protein and NF200 as an index for status of axons (spinal cord) of control and EAE animals (Fig 5A–5F). Animals treated with AKP-11 and FTY720 had reduced CNS infiltrating T cells when compared to control group as measured on H&E stain and CD4 mRNA analysis (Fig 5A–5C). To further investigate myelin specific Th17 cell infiltration in CNS, we isolated mononuclear cells from spinal cord and stained for CD4 and IL-17. The flow cytometric analysis revealed that there was a reduction in the number of Th17 cells in AKP-11 and FTY720 treated animals (Fig 5G). To analyze direct effects of AKP-11 and FTY720 on IL-17 production under Th17 skewed (rIL-6 and rTGF-β) conditions, purified spleen CD4 T cells were stimulated with CD3 and CD28 antibodies in the presence or absence of AKP-11 or FTY720 for 72hrs and IL-17 expression level in the media was analyzed. Both AKP-11 and FTY720 do not suppress IL-17 production (Fig 5H), indicating that AKP-11 and FTY720 inhibits egression of T cells from secondary lymphoid organs. We also investigated the efficacy of AKP-11 and FTY720 against EAE disease mediated neurodegeneration. For neurodegeneration studies, the spinal cord sections from control and experimental animals treated for 14 days starting at disease onset (Fig 1) were investigated for morphological analysis using Luxol Fast Blue (LFB) stain and biochemical analysis for MBP and neurofilament protein (NF 200). Fig 5C shows loss of MBP as well as NF200 in untreated EAE animals and treatments with AKP-11 or FTY720 provided protection against EAE disease induced loss of these proteins. Moreover, Fig 5D–5G show that inhibition of infiltrating immune cells into CNS with AKP-11 as well as FTY720 treated EAE animal decreased levels of their effector molecules such as inflammatory cytokines (IFN-γ and IL-17). However, in agreement with previous studies with FTY720 [39], treatments with AKP-11 or FTY720 has relatively little effect on expression of anti-inflammatory cytokines (IL-10). These data provides evidence that the inhibition of lymphocyte egression by AKP-11 as well as FTY720 results in reduced CNS infiltration of immune cells and decreased expression of pro-inflammatory cytokines and thus decreased neurodegeneration in EAE.

### AKP-11 decreases cell surface expression of S1P1 in *in vitro* cell culture model

**Fig 6:**
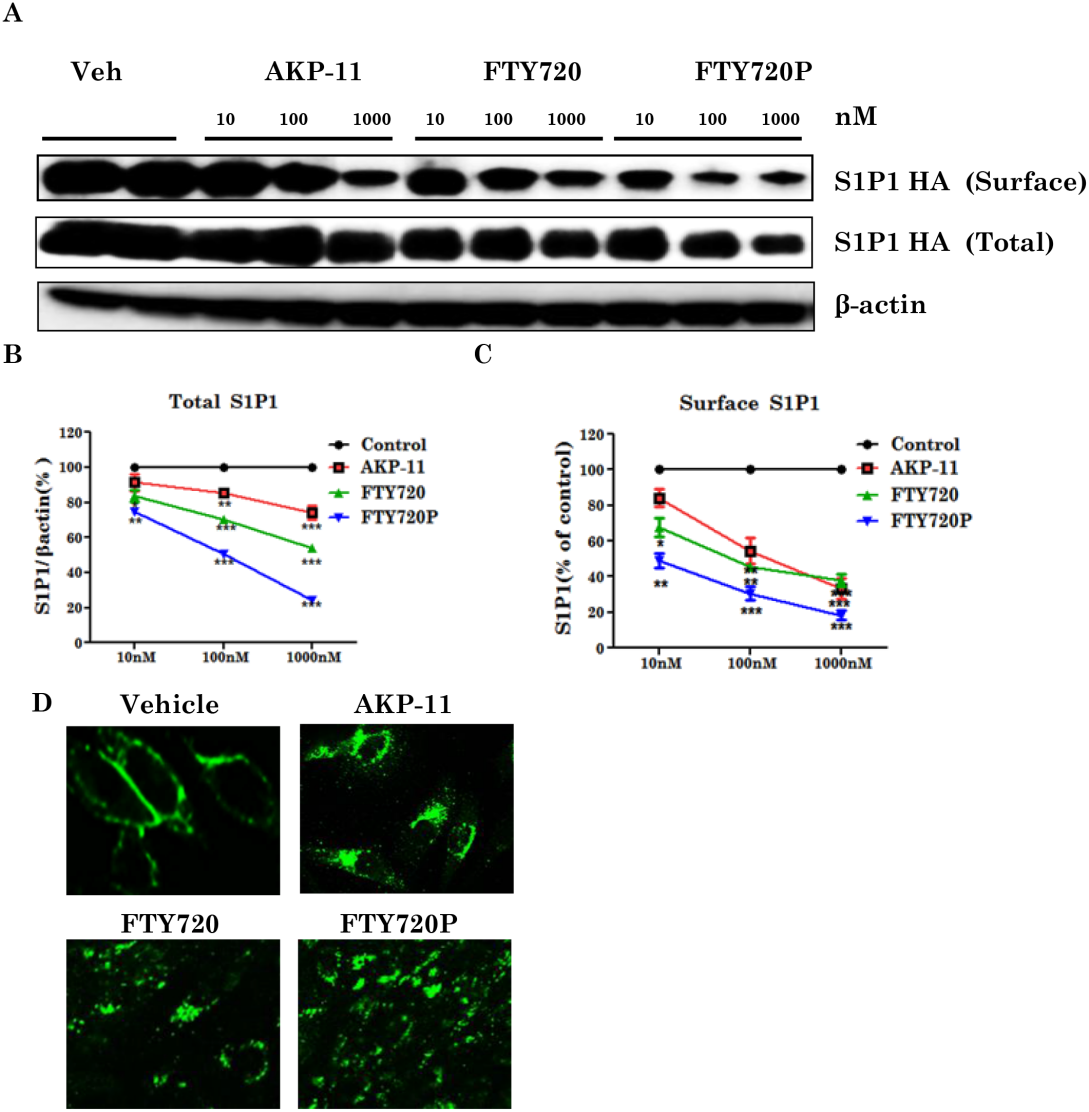
AKP-11 decreases cell surface expression of S1P1 receptor. (A-C) CHO cells were stably transfected with S1P1-HA constructs and treated for 2hrs with 10 or 100 or 1000nM of AKP-11 or FTY720 or FTY720P. Cell surface S1P1 HA was carried out by biotinylation method. (D) Confocal immunofluorescence analysis of surface S1P1 in CHO-S1P1-HA stable cells. 1000nM of AKP-11 or FTY720 or FTY720P were treated for 1hr. and fixed and immuno-stained with HA antibody. Data represents mean ± SEM of three independent experiments. Statistical significance indicated as *p<0.05 **p<0.01 and ***p<0.001.

**Fig 7:**
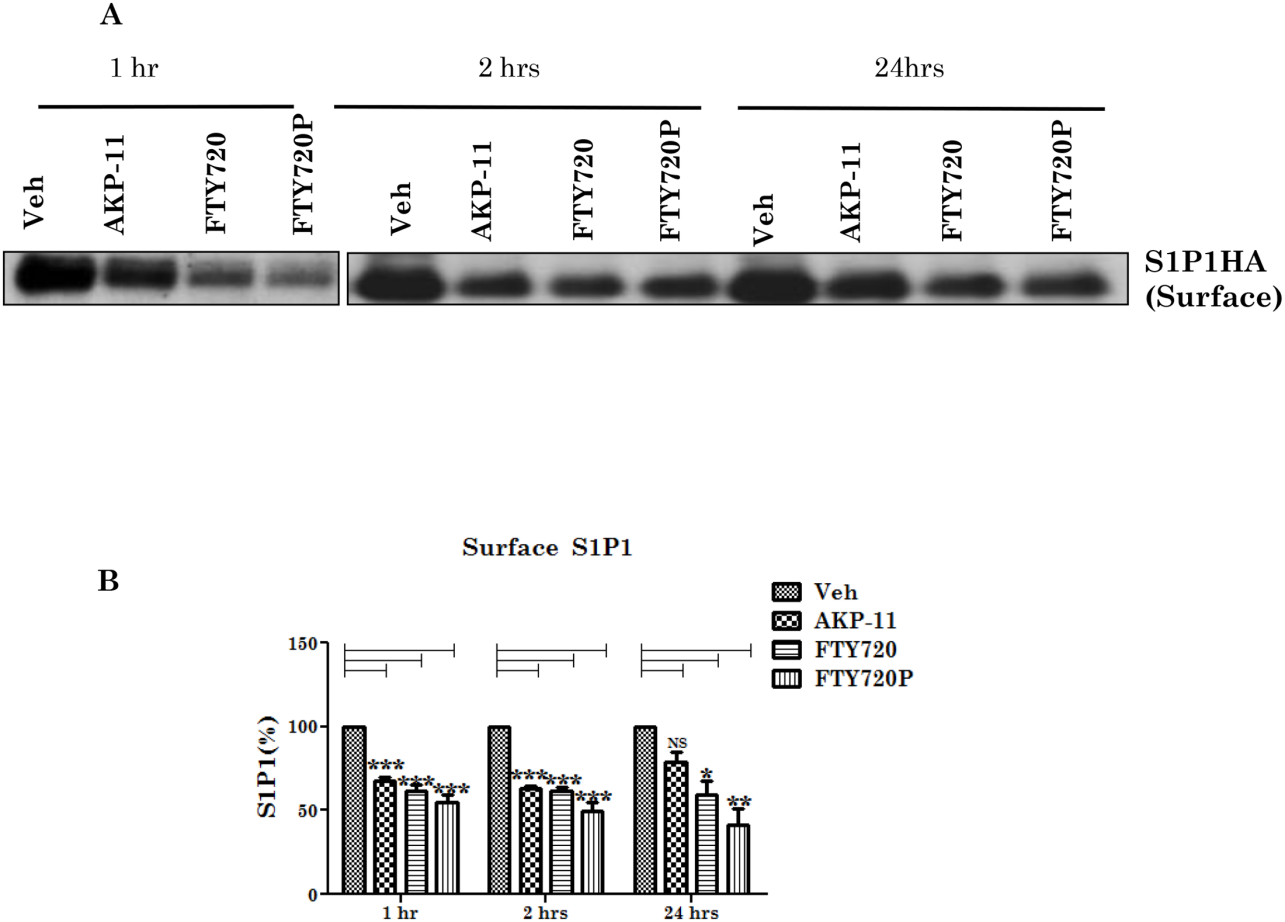
AKP-11 withdrawal increases cell surface S1P1 receptor expression. (A-B) CHO cells expressing S1P1-HA were pretreated with cycloheximide (15μg/ml) for 30 min to block the synthesis of new S1P1 and then stimulated with 100 nM AKP-11 or FTY720 or FTY720P for 1 hr. The above compounds were washed out and replenished with fresh serum free medium containing 0.5% fatty acid free BSA and cycloheximide and incubated for 2 or 24 hrs. Data represents mean ± SEM of three independent experiments. Statistical significance is indicated as *p<0.05 **p<0.01 and ***p<0.001, NS- not significant.

FTY720 as a S1P1 agonist is known to reduce cell surface distribution of S1P1 by its increased internalization and degradation [44–46]. In fact, efficacy of FTY720 in MS is due to S1P1 loss on lymphocytes leading to lymphopenia [14]. Since AKP-11 treatment causes relatively milder and rapidly reversible lymphopenia, effects of AKP-11 or FTY720 on S1P1 were investigated for S1P1 trafficking in stably expressing S1P1 CHO cells (CHOHA S1P1 cells) as described in methods section. CHO HA S1P1 cells were treated with different concentrations of AKP-11, FTY720 or FTY720P for 2hrs and levels of S1P1 in plasma membrane (surface) and total cellular levels of S1P1 were determined as described in methods section. Treatment with AKP-11 or FTY720 significantly reduced S1P1 on the plasma membrane indicating that both drugs induce internalization of S1P1 distribution on cell membrane (surface) (Fig 6A–6C). Treatment of FTY720 or FTY720P 100 and 1000nM for 2hr also reduced the total S1P1 levels indicating relatively higher degradation S1P1 in FTY720 and FTY720P as compared to AKP-11 treated cells (Fig 6B). Total S1P1 and plasma membrane S1P1 was reduced to a greater degree in FTY720 and FTY720P treated cells than in AKP-11 treated cells. These conclusions were further supported by the observed greater loss of immunofluorescence labelled S1P1 receptor in FTY720 and FTY720P treated cells as compared to AKP-11 treated cells (Fig 6D). These immunoctyochemical studies showed greater retention of membrane S1P1 and total S1P1 in AKP-11 treated cells. Next, we investigated recycling of S1P1 to the plasma membrane following withdrawal of drug treatment. For this study, cells were treated with AKP-11, FTY720 or FTY720P (100nM) for 1hr followed by change of media to remove the drug exposure to cells and cells were then incubated for 2 and 24hrs. As shown in Fig 7 there was a decrease in the amount of S1P1on the surface after 1hr treatment with AKP-11, FTY720 and FTY720P. At 2hrs following withdrawal with AKP-11 or FTY720 or FTY720P and FTY720P treatment caused a greater decrease of S1P1. At 24hr after drug withdrawal plasma membrane distribution of S1P1 increased in AKP-11 treated cells but not in FTY720 or FTY720P treated cells. These observations indicate that S1P1 recycles back following withdrawal of AKP-11 treated cells but not in FTY720 and FTY720P treated cells (Fig 7A and 7B). These studies are consistent with the observed milder and quickly reversible lymphopenia in AKP-11 treated animals as compared to FTY720 treated animals (Fig 4).

### Effects of AKP-11 or FTY720 on S1P1 internalization and degradation

**Fig 8:**
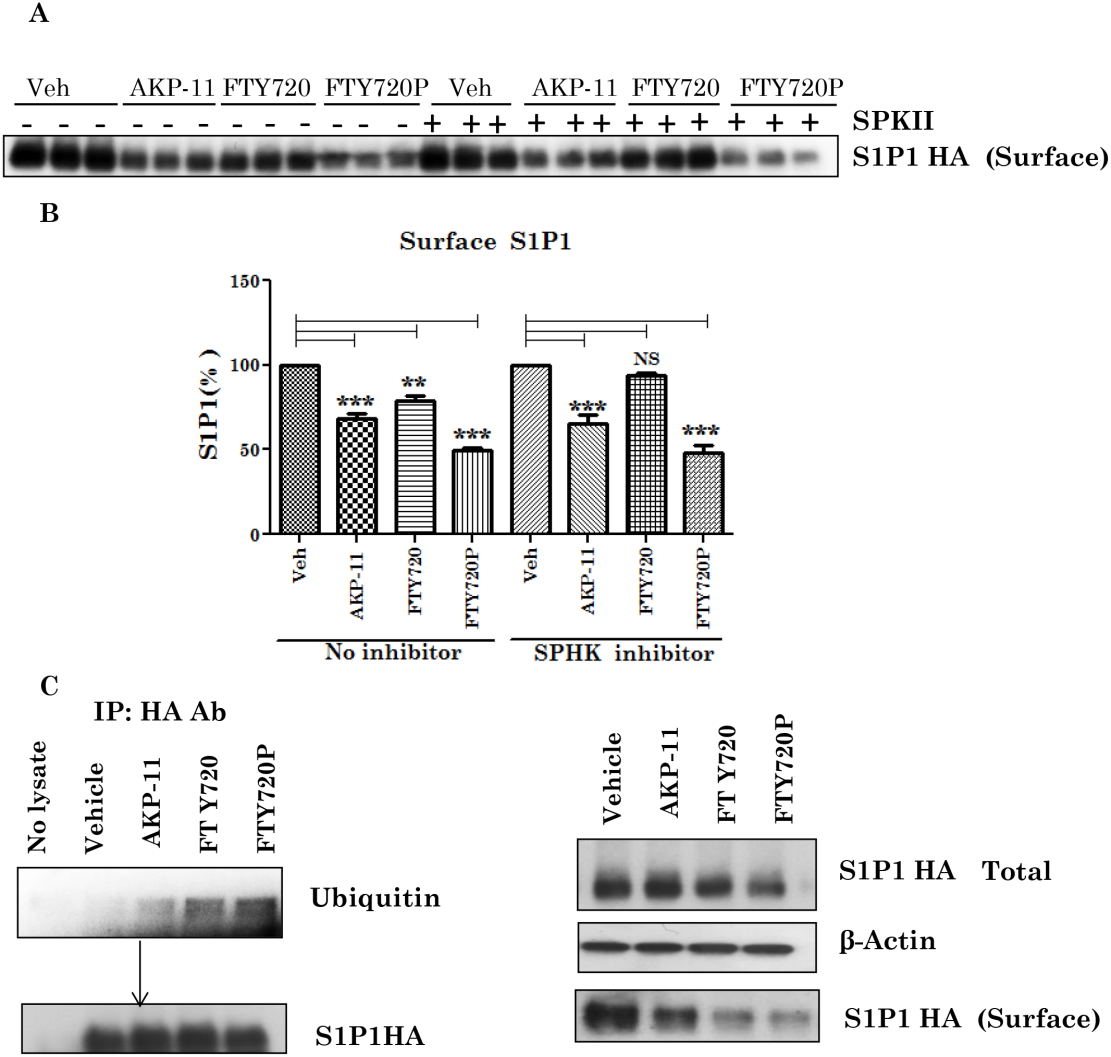
AKP-11 mediated S1P1 down-regulation is Sphingosine kinase independent and AKP-11 induces less ubiquitinylation. (A-B) 10μM of SPKII, a dual sphingosine kinase inhibitor treated in CHO-S1P1-HA stable cells for 30 min. After that, the cells were stimulated with 100 nM AKP-11 or FTY720, or FTY720P for 1 hr. Biotinylated cell surface S1P1 HA proteins were immunoblotted and quantitated. (C) CHO-S1P1-HA stable cells were pretreated 20μM MG132 for 2hrs and then stimulated with 1000 nM AKP-11 or FTY720, or FTY720P for 1hr. Cell lysates were immunoprecipitated with HA antibody and ubiquitination of S1P1 was detected by western blotting with ubiquitin antibody. The membrane was re-probed with HA antibody. (D) At the same time input lysates were detected by direct western blot with S1P1 HA antibody. Data represents mean ± SEM of three independent experiments. Statistical significance is indicated as *p<0.05 **p<0.01 and ***p<0.001, NS- not significant.

**Fig 9:**
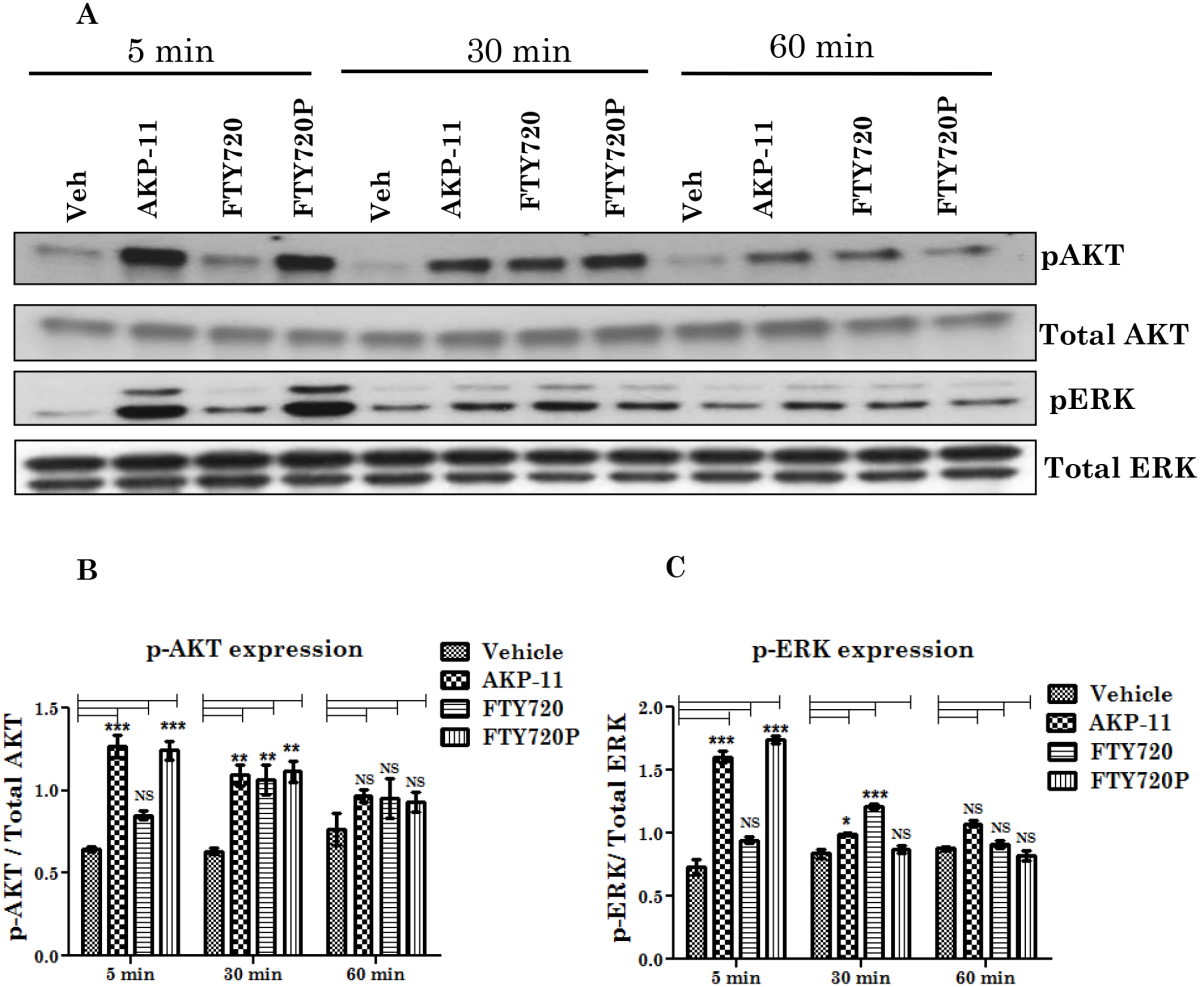
AKP-11 increases AKT and ERK activation through S1P1 receptor signaling. (A-C) CHO-S1P1-HA stable cells were stimulated with 100nM AKP-11 or FTY720, or FTY720P at different time points. (5 min, 30 min and 60 min) and were subjected to western analyses of phospho and total AKT and ERK proteins were quantitated. Data represents mean ± SEM of three independent experiments. Statistical significance is indicated as *p<0.05 **p<0.01 and ***p<0.001, NS- not significant.

FTY720 is a prodrug and is activated via its phosphorylation by sphingosine kinase, whereas FTY720P directly activates S1P1 signaling [17–19]. To evaluate the activity of AKP-11, cells were treated with dual sphingosine kinase inhibitor (SPKII) for 30 min followed by treatment with S1P1 agonists (AKP-11, FTY720 or FTY720P) for 1hr and plasma membrane distribution of S1P1 was investigated as described in methods. Interestingly, SPKII inhibitor treatment had no effect on the levels of S1P1 membrane distribution in AKP-11 or FTY720P treated cells (Fig 8A and 8B). These observations indicate that similar to FTY720P, AKP-11 binding to S1P1 and its internalization is independent of SPKII (Fig 8A and 8B). On the other hand, SPKII inhibitor prevented FTY720 but not FTY720P mediated S1P1 receptor internalization. These findings support the conclusion that AKP-11 and FTY720P are direct agonists of S1P1 whereas FTY720 is a prodrug and that it needs to be activated by SPKII [17–19]. Binding of FTY720 to S1P1 induced irreversible internalization followed by ubiquitination-mediated degradation [46] and proteolysis [45]. Since the degree of internalization and S1P1 loss varied between FTY720 and AKP-11 treatments, we investigated the role of AKP-11 in S1P1 ubiquitinylation. CHO cells expressing S1P1 HA were treated with FTY720, FTY720P or AKP-11 and cell homogenates were immunoprecipitated for S1P1 using anti-HA antibody followed by western analysis for ubiquitin using antibody against ubiquitin. Fig 8C shows a greater degree of S1P1 ubiquitinylation in FTY720 as well as FTY720P treated cells as compared to cells treated with AKP-11. The greater degree of ubiquitinylation of S1P1 with FTY720/FTY720P is consistent with the observed greater degree of S1P1 degradation than with AKP-11 treatment (Figs 6 and 8). These observations indicate that binding of AKP-11 to S1P1 does not affect irreversible internalization of S1P1 as observed with FTY720/FTY720P [45–47]. These conclusions are consistent with data in Fig 9 showing recycling of S1P1 to cell membrane following withdrawal of these drug treatments.

### AKP-11 induces intracellular S1P1 mediated AKT and ERK activation

S1P1 receptor agonists are known to activate AKT and ERK cellular signaling mechanisms [48,49]. Activation of AKT and ERK results in phosphorylation of Ser473 in AKT and Thr202/Tyr204 phosphorylation in ERK1/2, respectively. As shown in Fig 9, treatment of cells with AKP-11 or FTY720/FTY720P significantly increased pAKT and pERK levels when compared to untreated cells. AKT and ERK phosphorylation peaked after 5 minutes in cells of FTY720P or AKP-11 treatment but not in those treated with FTY720, a prodrug. It is only after 30 minutes of treatment with FTY720 that pAKT and pERK reached the levels observed with AKP-11 or FTY720P treatments for 5 minutes. There was also no difference in AKT and ERK phosphorylation at 60 min following treatment with AKP-11 or FTY720 compared to controls (Fig 9A–9C). The similar activities of FTY720P and AKP-11 at short term activation (5min) as compared to FTY720 indicate that similar to FTY720P, AKP-11 is direct agonist of S1P1. These conclusions are also supported by data in Fig 9 showing AKP-11 mediated internalization of S1P1 does not require its activation by sphingosine kinase II. On the other hand, FTY720 is a prodrug and needs to be phosphorylated by sphingosine kinase II [17–19].

### Role of FTY720 or AKP-11-mediated activation of S1P1 on lung vascular permeability and on heart rate

**Fig 10:**
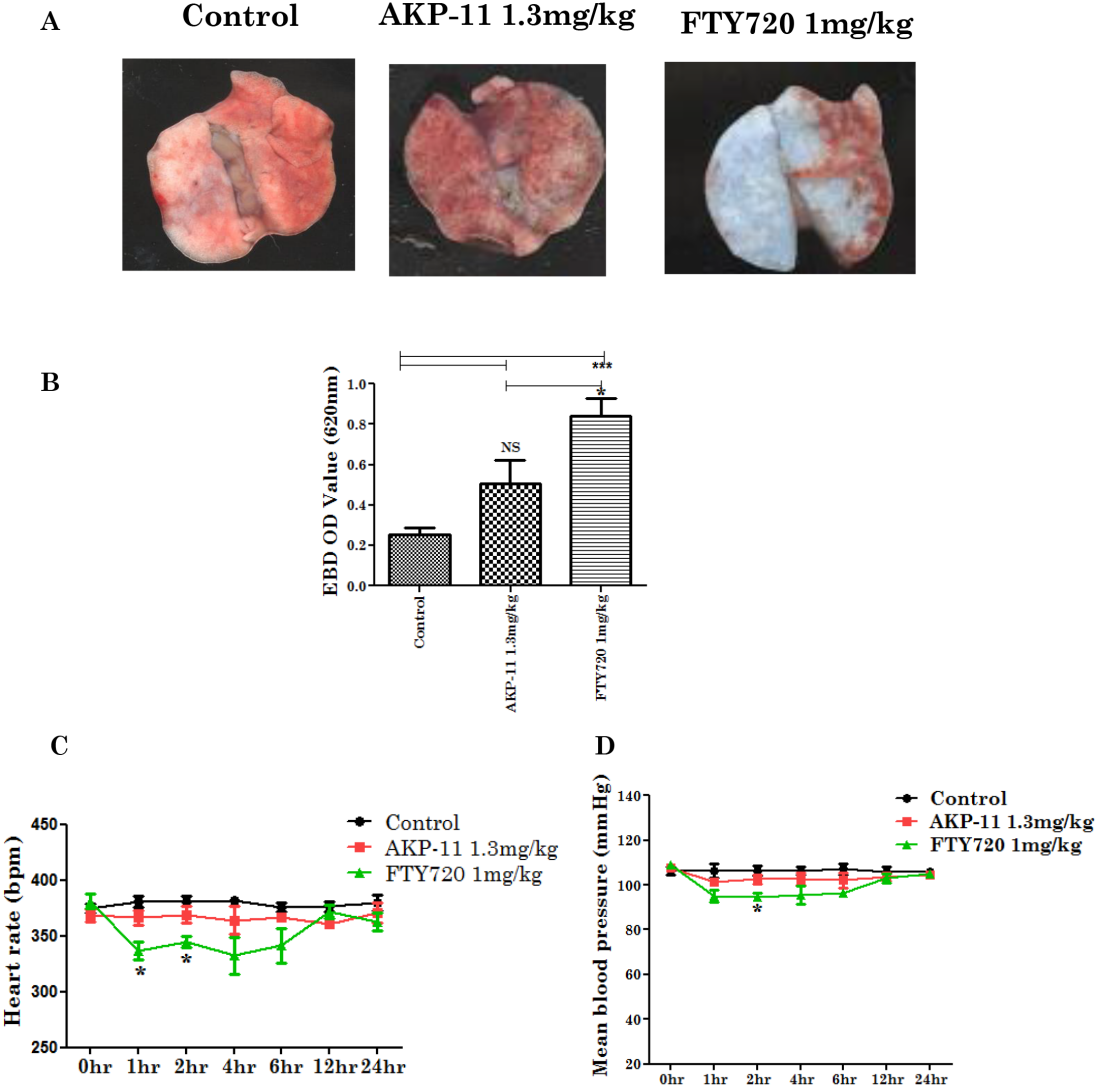
Effect of AKP-11 and FTY720 on lung vascular permeability and heart rate. (A-B) AKP-11 (1.3mg/kg) and FTY720 (1mg/kg) were orally administered. After 24hrs, Evans blue dye (EBD) was injected through tail vein and after 2hrs the animals were perfused with saline and lungs were photographed. EBD was measured in the lungs after extraction in the dimethylformamide solution. (C-D) Heart rate and blood pressure were measured at 0,1,2,4,6,12,and 24hr post oral administration of vehicle, AKP-11 (1.3mg/kg) and FTY720 (1mg/kg). Data represents mean ± SEM of three independent experiments (6 animals per group). Statistical significance is indicated as *p<0.05 **p<0.01 and ***p<0.001, NS- not significant.

FTY720 mediated lymphopenia is associated with a number of adverse effects [42]. Loss of S1P1 receptor is reported to increase vascular permeability in lungs [16,30]. We investigated the effect of AKP-11or FTY720 on lung vascular permeability by Evans blue dye extravasation assay as described under methods and materials. Fig 10A and 10B shows increased vascular permeability of Evans blue dye in lungs treated with FTY720 as compared to AKP-11. FTY720 treatment increased dye permeability by 4 fold whereas AKP-11 treatment increased the vascular permeability about 2 fold as compared to untreated controls. These observations indicate that AKP-11 causes less vascular dysfunction as compared to FTY720. Secondly, bradycardia is another major adverse effect caused by FTY720 [29,50,51]. Therefore, we also investigated the heart rate following single dose of AKP-11 (1.3mg/kg) and FTY720 (1mg/kg). AKP-11 treatment had little effect on the heart rate of animals. On the other hand, FTY720 treated animals had a significant drop (p<0.05) in heart rate following drug treatment (Fig 10C). Moreover, the decrease in blood pressure was also smaller with AKP-11 treatment as compared animals treated with FTY720 (Fig 10D). Consistent with previous findings, the observed mild and reversible lymphopenia with no effects on heart rate and relatively little changes in lung vascular integrity in animals treated with AKP-11 as compared to FTY720 indicate that AKP-11 has a favorable safety profile.

## Discussion

This manuscript describes the activities of a novel oral S1P1 agonist (AKP-11) with therapeutic efficacy similar to the one observed with FDA approved oral drug FTY720 in an animal model of MS but with a better safety profile as compared with FTY720. These conclusions are based on the following observations: 1) FTY720 and AKP-11 provide similar efficacy against clinical disease of EAE and protection against EAE disease induced neurodegeneration. 2) Both FTY720 and AKP-11, as S1P1 agonists induce similar cellular mechanisms such as activation of AKT and ERK signaling pathways. 3) Both FTY720 and AKP-11 provide efficacy against EAE via inhibition of S1P1 mediated lymphopenia and hence decreased infiltration of activated immune cells into the CNS. However, lymphopenia induced by AKP-11 was milder and transient (quickly reversible) as compared to the one induced by FTY720. 4) Accordingly, FTY720 treatment caused a greater degree of internalization, ubiquitination and degradation of S1P1 and thus loss of S1P1 recycling to plasma membrane when compared to AKP-11 treatment. 5) Consistent with previous reports on FTY720 treatment cause serious adverse effects [29,30,50,51] whereas AKP-11 treatment of rodents produced much milder adverse effects of lymphopenia, bradycardia and lung vascular leaks when compared to FTY720. We observed milder adverse effects while maintaining similar efficacy against the clinical disease of EAE documenting the favorable safety profile of AKP-11.

With the approval of fingolimod (FTY720), a S1P1, 3–5 agonist, as a first oral medication for the treatment of RRMS patients, S1P receptors have attracted a greater degree of interest for their contribution in various disease conditions [52]. S1P1-5 receptors are expressed on diverse cell types, belonging to a superfamily of G protein coupled receptors and are coupled to various cellular signaling activities [16,19,53]. FTY720, an analogue of sphingosine, is a prodrug and for its activity FTY720 is converted to phosphorylated FTY720 (FTY720P), an analogue of S1P and thus an agonist of S1P1, S1P3, S1P4 and S1P5 receptors [19]. S1P1-5 receptors participate in a wide range of cellular activities in lymphocytes [17,19], oligodendrocytes [54], endothelial cells [18], macrophages [55], astrocytes [56] and neurons [57]. FTY720 binding to S1P receptor leads to its irreversible internalization and degradation results in loss of its recycling to the membrane [46]. One of the important functions of S1P1 is S1P-dependent egress of lymphocytes from secondary lymphoid organs. The loss of lymphocyte cell surface S1P receptor 1 leads to loss of their response to S1P gradient that results in loss of lymphocyte egress from secondary lymphoid organs and thus lymphopenia [19,22]. In fact, FTY720 induced lymphopenia [58,59] is the basis of FTY720 as limited access of inflammatory lymphocytes into CNS and thus limited CNS disease process in EAE and MS. However, long lasting lymphopenia associated with loss of FTY720/S1PR functions alters cellular activities leading to adverse effects [31,60]. In contrast, studies described in this manuscript document that similar to FTY720, AKP-11 functions as a S1P1 agonist (Figs 2–5) and activation of cellular AKT and ERK signaling pathways (Fig 9). However the rate of internalization and degradation of S1P1 was much greater with FTY720 than AKP-11 (Fig 6). AKP-11 treatment of control and EAE animals causes relatively milder and reversible lymphopenia (Figs 2–4) suggesting possible limited milder adverse effects with AKP-11 as compared to FTY720. In this study, total lymphocytes in EAE animals is similar or lower than that of control animals due to lymphocytes that were counted on remission condition at day 18 and 26 post immunization. Our data is consistent with previous study by Webb et al [61] that showed an increase in blood lymphocytes at early stage of EAE than control and then decreased during the chronic phase of the disease.

FTY720 is a prodrug and is phosphorylated by SPHK2 in vivo and phosphorylated FTY720 (FTY720P) interacts and activates S1P1 surface receptors [17,19,45]. The role of SPHK2 in FTY720 drug action was supported by lack of lymphopenia in SPHK2 knockout mice with FTY720 whereas administration of FTY720P caused lymphopenia in this knockout model [62]. The present study showing lack of effect of SPHK2 activity on AKP-11 mediated internalization and activity of S1P1 documents that AKP-11 is not a pro-drug, rather it is a direct agonist of S1P1 receptor (Fig 8) Recently, two other S1P1 agonist butterfly compounds ST-968 and ST-107 were also shown to be independent of SPHK2 activity in inducing S1P1 receptor internalization and reducing clinical disease in EAE mice [63]. The AKP-11 activity seems to be similar to S1P showing recycling of S1P1 to the plasma membrane [44]. The reduced ubiquitination of S1P1 and degradation and the increased recycling of S1P1 with AKP-11 as compared to FTY720 or FTY720P following withdrawal of AKP-11 (Figs 7 & 8) could possibly be basis of milder and reversible lymphopenia effect observed with AKP-11. The higher levels of S1P1 in AKP-11 as compared to FTY720 treated cells is in agreement with the observed greater ubiquitination of S1P1 and turnover in FTY720 treated cells. AKP-11 as well as FTY720 or FTY720P treatments decreases the distribution of S1P1 in the cell membrane (Fig 6) but it cycles back to membrane only in AKP-11 treated cells as observed by higher levels of S1P1 at 24hrs as compared to 2hrs post treatment (Fig 7). On the other hand, the loss of cell membrane distribution of S1P1 in FTY720 or FTY720P treated cells didn’t change with time. These studies describe the activities of a novel S1P1 agonist (AKP-11) that produces milder and reversible lymphopenia as compared to FTY720 producing a greater loss of S1P1 and thus prolonged lymphopenia. CNS disease of MS/EAE initiates with the infiltration of myelin specific immune cells and expression of inflammatory mediators (TNFα, IL-1β, IFN- γ and IL-17), demyelination and axonal degeneration leading to physical disability observed in patients with MS [64–66]. Oral administration with AKP-11 following the onset of EAE clinical disease protected against EAE disease progression. Histological studies show reduced infiltration of inflammatory cells into the CNS of animals treated with AKP-11 or FTY720 resulting in reduced demyelination and axonal degeneration assessed by the levels of MBP and NF200 proteins (Fig 5). Our data shows decreased CD4^+^ T cell infiltration into the spinal cord of AKP-11 and FTY720 treated EAE animals (Fig 5B and 5G). The CD4^+^IL-17^+^ cells increased in the CNS of EAE animals, reflect trafficking of myelin antigen specific T cells into the CNS and promotes development and progression of EAE. AKP-11 and FTY720 has no effect on Th17 response (Fig 5H). Both these compounds reduce S1P responsiveness and inhibit the lymphocyte egress from secondary lymphoid organs. Accordingly, FTY720 has no effect on IL-17 production in in-vitro activated human T cells [67].The reduced infiltration of inflammatory T cells into the CNS (Fig 5A–5B and 5G) is consistent with decreased egress of lymphocytes (CD4, CD8 and CD62L T cells) (Figs 2–4). Consistent with the transient loss of S1P1 with AKP-11 as compared to FTY720 treatment (Fig 6), AKP-11 caused milder (Figs 2 and 3) and transient lymphopenia (Fig 4) as compared to prolonged lymphopenia with FTY720. In spite of the milder lymphopenia observed with AKP-11, equimolar dose of AKP-11 and FTY720 provided similar degrees of therapeutic efficacy against the clinical disease of EAE (Fig 1) as well as in the protection against the EAE disease induced neurodegeneration (Fig 5C).

FTY720 is an attractive drug for oral medication however it suffers from significant adverse effects such as lymphopenia, bradycardia [42,68] and vascular effects [30,31]. Recent reports have described a number of S1P1 agonists and antagonist as a substitute for FTY720, however, a number of them have not studied their activities relative to the above adverse effects [63,69,70] while others were reported for one or other adverse effects [68,71]. Some of these studies also need comparison against FTY720. The milder and reversible lymphopenia with AKP-11 as compared to FTY720 suggest that AKP-11 may be a safer drug. In our study, we tested the oral administration of equimolar dose of AKP-11 or FTY720 for clinical efficacy against EAE disease as well as on adverse effects of reversibility of lymphopenia, heart rate and lung vascular leaks in the rat model. As shown in Fig 10, FTY720 treatment caused significant change in vascular leaks (Fig 10A and 10B) and drop in heart rate (Fig 10C) whereas AKP-11 treatment caused relatively milder vascular leaks and an insignificant change in heart rate. The above observations indicate that AKP-11 as compared to FTY720 and other S1P1 agonists has a favorable safety profile. FTY720 induced bradycardia caused by activation of S1P3 receptor, is supported by lack of bradycardia in S1P3 knock out mice [72,73]. Moreover, inhibition of S1P3 activation with its antagonist (TY-52156) prevented FTY720 induced bradycardia [74].

In summary, this study describes the properties of a novel S1P1 agonist AKP-11 using cell culture and animal model of EAE. Both treatments of EAE animals with AKP-11 or FDA approved drug for MS (FTY720) provided similar efficacy and neuroprotection in the EAE model. AKP-11 treatment caused milder and reversible lymphopenia as compared to the severe and long lasting lymphopenia observed with FTY720. Secondly, AKP-11 treatment did not cause adverse effects observed with FTY720 treatment. These findings indicate that AKP-11 mediated S1P1 agonist activity may be of therapeutic value for MS and other immune mediated disorders with better safety profile.
